# Comparison of disc diffusion, Etest and broth microdilution for testing susceptibility of carbapenem-resistant *P. aeruginosa *to polymyxins

**DOI:** 10.1186/1476-0711-6-8

**Published:** 2007-08-15

**Authors:** Inneke M van der Heijden, Anna S Levin, Ewerton H De Pedri, Liang Fung, Flavia Rossi, Gisele Duboc, Antonio A Barone, Silvia F Costa

**Affiliations:** 1 Laboratory of Medical Investigation 54, Hospital das Clinicas, University of São Paulo, Brazil; 2 Department of Infectious, Diseases of University of São Paulo, Brazil; 3 Laboratory of Microbiology of Hospital das Clínicas, University of São Paulo, Brazil; 4 Fernão Dias 158 apt 71, Pinheiros, São Paulo, SP, Brazil

## Abstract

**Background:**

Considering the increasing use of polymyxins to treat infections due to multidrug resistant Gram-negative in many countries, it is important to evaluate different susceptibility testing methods to this class of antibiotic.

**Methods:**

Susceptibility of 109 carbapenem-resistant *P. aeruginosa *to polymyxins was tested comparing broth microdilution (reference method), disc diffusion, and Etest using the new interpretative breakpoints of Clinical and Laboratory Standards Institute.

**Results:**

Twenty-nine percent of isolates belonged to endemic clone and thus, these strains were excluded of analysis. Among 78 strains evaluated, only one isolate was resistant to polymyxin B by the reference method (MIC: 8.0 μg/mL). Very major and major error rates of 1.2% and 11.5% were detected comparing polymyxin B disc diffusion with the broth microdilution (reference method). Agreement within 1 twofold dilution between Etest and the broth microdilution were 33% for polymyxin B and 79.5% for colistin. One major error and 48.7% minor errors were found comparing polymyxin B Etest with broth microdilution and only 6.4% minor errors with colistin. The concordance between Etest and the broth microdilution (reference method) was respectively 100% for colistin and 90% for polymyxin B.

**Conclusion:**

Resistance to polymyxins seems to be rare among hospital carbapenem-resistant *P. aeruginosa *isolates over a six-year period. Our results showed, using the new CLSI criteria, that the disc diffusion susceptibility does not report major errors (false-resistant results) for colistin. On the other hand, showed a high frequency of minor errors and 1 very major error for polymyxin B. Etest presented better results for colistin than polymyxin B. Until these results are reproduced with a large number of polymyxins-resistant *P. aeruginosa *isolates, susceptibility to polymyxins should be confirmed by a reference method.

## Background

Polymyxins are a multicomponent polypeptide antibiotic that act primarily on the gram-negative bacterial cell wall, leading to rapid permeability changes in the cytoplasmic membrane and ultimately to cell death [1]. The polymyxin E named colistin and polymyxin B have been used to treat several infections caused by multidrug resistant *Pseudomonas aeruginosa *(MDR-PA) isolates, which are resistant to aminoglycosides, cephalosporin and penicillins anti-pseudomonas, quinolones, monobactams and carbapenem [2,3]. Our hospital has been using polymyxins as a therapeutic option to treat MDR-PA infection since an outbreak that occurred in 1992 [2,4,5].

Although the availability of less toxic antipseudomonal antibiotics relegated polymyxins to the status of a reserve agent, the subsequent development of MDR-PA has made polymyxins of interest once more, as it possess the advantage of rapid bactericidal activity and only slowly leads to the development of resistance [3,6,7].

Only in 2005 the National Committee on Clinical Laboratory Standards (NCCLS) now called Clinical and Laboratory Standards Institute (CLSI), approved a standard document for the testing of polymyxins using dilution method [8]. However, disc susceptibility testing methods remain the most commonly used techniques in clinical microbiology laboratories. Until recently the zone size interpretations were based on data from literature [9]. Interpretative criteria for disc susceptibility testing of polymyxin were this current year available from the CLSI [10]. European guidelines for polymyxin E disc susceptibility testing were also published by the British Society for Antimicrobial Chemotherapy (BSAC), the Société Francaise de Microbiologie (SFM) and the German Deutsches Institut fur Normung (DIN) [11,12]. However, there is not a consensus regarding the breakpoint to define resistance to polymyxins.

Considering the increasing use of polymyxins to treat infections due to multidrug resistant Gram-negative infections in many countries, it is important to evaluate different susceptibility testing methods to this class of antibiotic.

In this study, the antimicrobial activities of the polymyxins were evaluated against carbapenem-resistant *P. aeruginosa *isolated from blood over a six-year period comparing different assays such as broth microdilution, disc diffusion, and Etest using the new CLSI criteria [10].

## Methods

A total of 109 strains of *P. aeruginosa *isolated from patients with bloodstream infection (BSI) over a 6-year period (1998–2003), were identified by Vitek (BioMérieux) and this identification was confirmed by classical microbiological testing methods. Carbapenem-resistance was defined as: isolates resistance to imipenem or meropenem by broth microdilution susceptibility testing.

### 2.1 Susceptibility test

Polymyxin B and colistin sulfate powders were obtained from Sigma Chemical (St. Louis, Mo.). One mg of polymyxin B is equivalent to 8,240 units and 1 mg of polymyxin E to 19,530 units. The other tested drugs were obtained commercially or provided by their respective manufacturers.

Susceptibility to the following antibiotics was determined by broth microdilution method: imipenem, meropenem, cephalosporins and penicillins anti-pseudomonas, quinolones, monobactam and aminoglycosides [13]. Polymyxins susceptibility testing was performed using disc diffusion, broth microdilution and Etest (AB Biodisk) methods. Disc diffusion was done using 10 μg colistin disc (Oxoid), and 300 U polymyxin B disc (Oxoid). Broth microdilution with cation-adjusted Muller-Hinton broth (BBL-Becton Dickinson) was carried out in accordance with CLSI recommendations and was used as reference method [8,10]. The same inoculum was used for disc, Etest and broth microdilution testing. Bacterial suspensions were adjusted according to CLSI recommendations and the final inoculum was verified for all three susceptibility methods [13]. MIC_50 _and MIC_90 _were calculated for all antibiotics tested.

MICs were also determined by Etest method according to the manufacturer's guidelines (AB Biodisk, Solna, Sweden). MICs of Etest were rounded up to the next higher twofold dilution. MICs ≥ 8 μg/mL for polymyxin B and colistin were the breakpoint to designate resistant isolates for microdilution and Etest methods [14]. Disc zone diameters were interpreted according to the CLSI for colistin (resistant ≤ 10 mm and susceptible ≥ 11 mm) and for polymyxin B (resistant ≤11 mm and susceptible ≥12 mm) [10]. Agreement between Etest and microdilution was defined as MICs that differed by ± 1log_2 _dilution or less. Categorical agreement was defined as test results within the same susceptibility. Errors were ranked as follows: very major error, false-susceptible result by the disc diffusion test; major error, false-resistant result produced by the disc diffusion test; and minor error, intermediate result by disc diffusion method and a resistant or susceptible category for the reference method (microdilution test). Unacceptable levels are ≥ 1.5% for very major errors, ≥3% for major errors and 10% for minor errors as recommended in CLSI document M23-A2 [15].

*Escherichia coli *ATCC 25922 and *P. aeruginosa *ATCC 27853 were carried out as quality control (QC) strain for all susceptibility testing methods [10,16].

### 2.2 Molecular typing

The preparation of chromosomal DNA for PFGE was performed as described elsewhere. Bacterial isolates were grown on blood agar overnight at 37°C. Gel blocks were made by using equal volumes of 2% low-melting-point agar (BioRad) and a bacterial suspension of 9 × 10^8 ^cells. DNA was digested with Spe-I (New England BioLabs) at 37°C for 15 hours. The PFGE was performed with use of 1% agarose gel (BioRad) in a CHEF DRII system (BioRad) under the following conditions: run time, 20 hours; temperature, 14°C; voltage, 200 V; initial forward time, 5 s; final forward time 90 s; Lambda concatamers were run in the first and last lanes. Genotypes were defined on the basis of DNA banding patterns. Isolates with identical patterns were considered genotypically "indistinguishable", while those that differed by 1 to 3 bands were defined as "closely related" and 4 to 6 bands as "possible related". "Unrelated" or "different" strains referred to those that differed by ≥7 bands [17].

## Results

A total of 109 BSI carbapenem-resistant *P. aeruginosa *isolates obtained from 93 patients were studied. All isolates were resistant to imipenem and only two were susceptible to meropenem (MICs 2.0 μg/mL and 4.0 μg/mL). The colistin MIC_50 _and MIC_90 _were both 1.0 μg/mL and polymyxin B MIC_50 _and MIC_90 _were 0.5 and 1.0 μg/mL, respectively (table 1).

**Table 1:** Activities of ten agents against 109 isolates of carbapenem-resistant *P. aeruginosa *isolates obtained from patients with BSI over a six-year period.

Agent	MIC_50 _μg/mL	MIC_90 _μg/mL	Range μg/mL	% Susceptibility
Aztreonam	64	128	1 – >128	7.4
Cefepime	32	128	<0.25 – >128	7.3
Ceftazidime	128	>128	<0.25 – >128	8.3
Ciprofloxacin	32	64	<0.25 – 64	6.4
Colistin	1	1	<0.25 – 2	100
Gentamicin	>128	>128	0.5 – >128	20.2
Imipenem	64	256	16 – 1,024	0
Meropenem	32	256	2 – 1,024	1.8
Piperacillin/Tazobactam	>128	>128	1 – >128	13.8
Polymyxin B	0.5	1	<0.25 – 8	99.1

**Figure 1:**
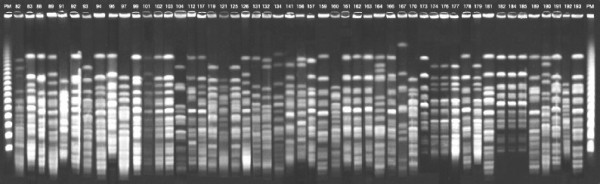
Pulsed-field gel electrophoresis results showing different clones of carbapenem-resistant *P. aeruginosa *compared with endemic clone represented by numbers 161, 162 and 163.

All strains were analyzed by PFGE method. We identified one endemic clone (clone A) in 29% of these isolates Figure 1. The clone A isolates were susceptible to both polymyxins (MICs ranged from 0.25 to 2.0 μg/mL). The MIC_50 _and MIC_90 _for colistin were 1.0 and 2.0 μg/mL, respectively. These isolates had the same value of MIC_50 _and MIC_90 _(1.0 μg/mL) to polymyxin B. So, strains that belonged to clone A were excluded.

**Figure 2:**
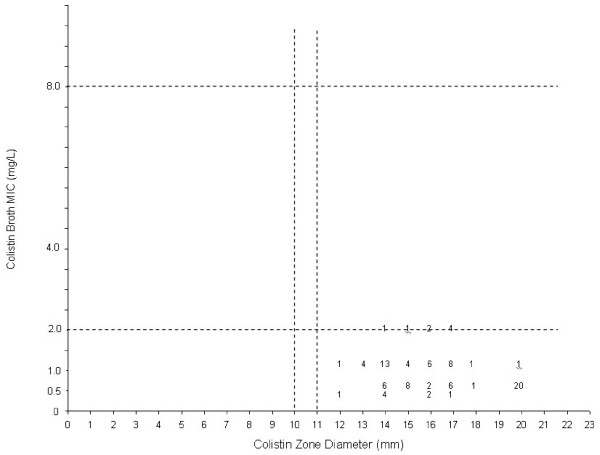
Comparative results between broth microdilution MICs and 10-μg disc zone diameters for Colistin tested against 78 carbapenem-resistant *P. aeruginosa *isolates. The broken lines represent the breakpoint values for Polymyxin E by disc diffusion method [10].

**Figure 3:**
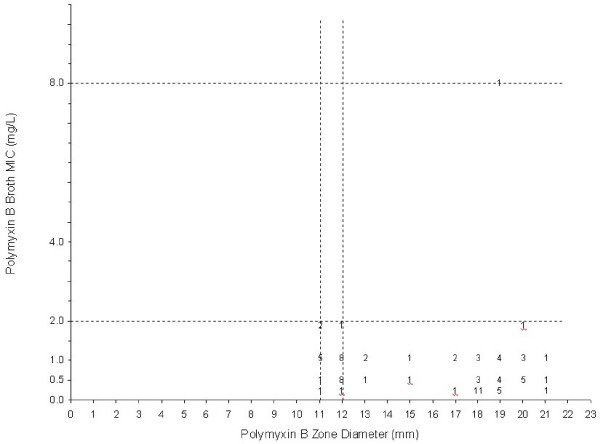
Comparative results between broth microdilution MICs and 300 U disc zone diameters for Polymyxin B (PB) tested against 78 carbapenem-resistant *P. aeruginosa *isolates. The broken lines represent the breakpoint values for Polymyxin B by disc diffusion method [10].

According to the breakpoints for disc diffusion testing by CLSI[10], 9 (11.5%) isolates were resistant and 69 (88.5%) susceptible to polymyxin B. However, only one isolate were defined as resistant to polymyxin B by broth microdilution (MIC 8.0 μg/mL) this strain had a MIC of 2.0 μg/mL to colistin. These results were confirmed by repeating it three times using the same procedure conditions. For colistin, all strains 78 (100%) were susceptible by disc diffusion and broth microdilution (Figures 2 and 3). Very major and major error rates of 1.2% and 11.5% were detected comparing polymyxin B disc diffusion with the broth microdilution (reference method) and no errors were detected with colistin.

**Figure 4:**
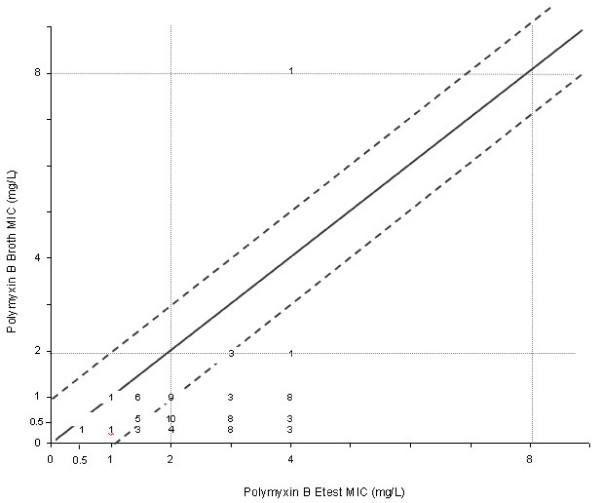
Scattergram results comparing polymyxin B MICs determined by Etest with those by the broth microdilution reference method (n = 78). The diagonal black line represents complete agreement, and the numbers represent the occurrences observed at each point. The broken lines represent ± 1-log_2 _MIC agreement limits between test results. Horizontal and vertical broken lines indicate the resistant MIC breakpoint ≥8 mg/L) [10].

**Figure 5:**
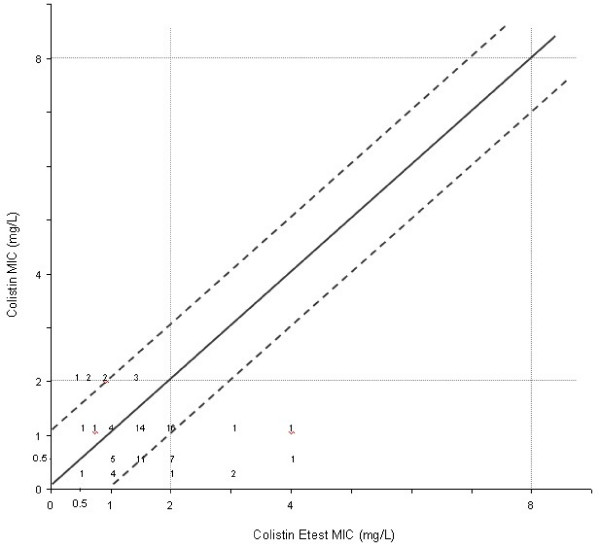
Scattergram results comparing colistin MICs determined by Etest with those by the broth microdilution reference method (n = 78). The diagonal black line represents complete agreement, and the numbers represent the occurrences observed at each point. The broken lines represent ± 1-log_2 _MIC agreement limits between test results. Horizontal and vertical broken lines indicate the resistant MIC breakpoint ≥8 mg/L) [10].

Agreement within 1 twofold dilution between Etest and the broth microdilution reference method were 33% for polymyxin B and 79,5% for colistin. The comparison between broth microdilution and Etest MICs results for polymyxin B and colistin are shown in (Figure 4 and 5). One (1,2%) very major error and 38 (48,7%) minor errors were found comparing polymyxin B Etest with microdilution and 5 (6,4%) minor errors with colistin. The cathegorical concordance was respectively 100% for colistin and 90% for polymyxin B (Table 2). The MICs determined by broth microdilution testing for the QC strains (*E. coli *ATCC 25922 and *P. aeruginosa *ATCC 27853) were the same as the range proposed by CLSI. The colistin disc zone diameters ranged from 15 to 20 mm for the *E. coli *ATCC 25922, and varied from 15 to 19 mm for *P. aeruginosa *ATCC 27853. For polymyxin B disk tests, the QC ranges were 17 to 20 mm for *E. coli *and 17 to 22 mm for *P. aeruginosa*. The Etest *E. coli *QC results ranged from 0.25 to 1.0 μg/mL for polymyxin B and from 0.125 to 0.5 μg/mL for colistin. For *P. aeruginosa *ATCC 27853 the QC results were 0.5 to 2.0 μg/mL for both polymyxins tested.

**Table 2:** Microdilution and Etest discrepancy rates for polymyxin B and Colistin and 78 carbapenem-resistant *P. aeruginosa *isolates.

Antimicrobial agents	No. (%) ^a^of discrepancies		
	Very Major Error	Major error	Minor
Polymyxin B	1 (1,2%)	_	38 (48,7%)
Polymyxin E	_	__	5 (6,4%)

^a^Unacceptable levels are show in bold. They are ≥ 3% for major errors as recommended in CLSI document M23 [15].

## Discussion

Emerging resistance in *P. aeruginosa *leads to the need for the parenteral use of polymyxins and reliable susceptibility methods to predict the clinical response. In this current year the CLSI recommended a new breakpoint and guidance for disc diffusion and microdilution methods for *P. aeruginosa *[10].

Polymyxins resistance among *P aeruginosa *isolates has been described in patients with cystic fibrosis, however, data on acquired resistance to polymyxins among isolates causing nosocomial infection are scanty [18,19]. The majority of our isolates remained susceptible to colistin despite the widely use of this drug in our hospital [4,5]. Only one isolate (1.2%) was resistant to polymyxin B (MIC 8.0 μg/mL) by broth microdilution method. Two recent studies in hospitals of New York showed similar results [20,21]. One study demonstrated that only 5% of 527 *P. aeruginosa *isolates from 11 hospitals had reduced susceptibility to polymyxin B with MICs ranging from 4 to 8 μg/mL. However, this study evaluated a short period of time of three months [20]. The other study did not find any isolate resistant to polymyxins [21]. The results of our study reinforce that polymyxins are still a good option to treat bloodstream infection due to carbapenem-resistant *P. aeruginosa*, and that the increase of resistance to polymyxins appears to be rare among the isolates from our hospital.

The disc diffusion technique was reported to be an unreliable method for evaluating the susceptibility to polymyxins [9,22]. Despite the recent recommendations of CLSI, data from Gales and colleagues using other breakpoint showed that disc diffusion assay for polymyxins are likely to be used only as a screening test to polymyxins susceptibility. Colistin displayed no good activity against *P. aeruginosa *isolates (MIC_90 _4 mg/L) and there were many false-susceptible results [9]. The accuracy of disc diffusion assays is unsatisfactory because polymyxins diffuse poorly into agar and consequently results of a diffusion test should be confirmed with a dilution method. In a recent study, the susceptibility to polymyxins of 228 clinical isolates of *Acinetobacter *sp., *P. aeruginosa *and Enterobacteriaceae was tested by agar dilution and the results were compared with those obtained by three standardized disc susceptibility testing methods (CLSI methodology, BSAC and SFM) [22]. These authors showed that disc diffusion produced an unacceptably high rate of very major errors (5–11%). Thus, according to this study disc diffusion remains an inherently unreliable susceptibility testing method for polymyxins [22]. Our study is the first one that used the new interpretative breakpoints for polymyxins recommend by the CLSI [10]. Frequent minors errors and 01 very major were finding with polymyxin B comparing disc diffusion with microdilution (reference method). Better results, however, were finding using colistin.

The breakpoints for polymyxins susceptibility differ among scientific communities. The SFM specifies that isolates with MIC >2 mg/L are considered resistant, while the BSAC adopts a breakpoint of >4 mg/L [11,12] and the CLSI published resistance breakpoints of ≥ 8 mg/L for *P. aeruginosa *[10]. The comparison of the three methods should be used cautiously since they differ in various parameters such as inoculum size and the medium used. Likewise, pH, anion content, effect of sulphomethyl derivatives comparing with sulphate and others characteristics can influence the results. The best reference method is not known and further studies need to address this point. However, there is a study that showed a good concordance between agar dilution and microdilution [23].

In the literature there are only few studies on the reliability of Etest as a method for susceptibility testing for polymyxins against Gram-negative. One study suggested that Etest could be an useful test as a screening method to detect polymyxin B and colistin resistance among *Stenotrophomonas maltophilia *isolates, with an agreement of 96.7% and 89.4%, respectively [24]. However, it showed a high frequency of major errors.

This is the first study that compared polymyxin B and colistin Etest with broth microdilution test (reference method) to detect susceptibility among carbapenem-resistant *P. aeruginosa *using the new breakpoints recommended by CLSI [10]. A recent study compared susceptibility to colistin of 172 isolates of Enterobacteriacae including 47 isolates of *P. aeruginosa *using Etest, Vitek and agar dilution. This study showed 11% of very major error and 30% of major error with Etest and 30% of very major errors with Vitek [25]. Goldstein et al [26] evaluated 170 clinical isolates of Gram-negative including a total of 64 *P. aeruginosa *(12 colistin-resistant strains) and compared Etest with agar dilution (reference method) for testing susceptibility to colistin. MICs of < 4 mg/l were considered to indicate susceptibility to colistin. Etest showed 91% of agreement (± 2-fold dilution) in comparison with the reference method. We observed a different result than these previous reports. The polymyxin B Etest showed unacceptable 38 (48,7%) minor errors and 1 (1,2%) very major error comparing with the broth microdilution test (the reference method), whereas colistin Etest had only 5 (6,4%) minor errors. Agreement within 1 twofold dilution between polymyxin E Etest and the broth microdilution was 79.5% and concordance of 100%. This result is similar to those described for *Acinetobacter *spp. by Arroyo et al [27], that showed a concordance of 98.2% of colistin Etest and the reference broth microdilution method, with only two (1.7%) very major errors.

A major problem with our study is the collection of strains used, since they were isolated only in one center and only 01 strain was resistant to polymyxin B. Nevertheless, the evaluation of clonality showed that only 29% of isolates belonged to the endemic clone (clone A) previously reported in our hospital and these strains were excluded of the comparison of analysis of susceptible methods.

The results of the present study showed using the new CLSI criteria that the disc diffusion does not report major errors (false-resistant) results for colistin. On the other hand, showed a high frequency of minor errors and 1 very major error for polymyxin B. Etest presented better results for colistin than polymyxin B. Until these results are reproduced with a large number of polymyxins-resistant *P. aeruginosa *isolates, susceptibility to polymyxins should be confirmed by a reference method.

## References

[B1] Evans ME, Feola DJ, Rapp RP (1999). Polymyxin B sulfate and colistin: old antibiotics for emerging multiresistant gram-negative bacteria. Ann Pharmacother.

[B2] Levin AS, Barone AA, Penco J, Santos MV, Marinho IS, Arruda EA, Manrique EI, Costa SF (1999). Intravenous colistin as therapy for nosocomial infections caused by multidrug-resistant *Pseudomonas aeruginosa *and *Acinetobacter baumannii*. Clin Infect Dis.

[B3] Littlewood JM, Koch C, Lambert PA, Hoiby N, Elborn JS, Conway SP, Dinwiddie R, Duncan-Skingle F (2000). A ten year review of colomycin. Respir Med.

[B4] Arruda EA, Marinho IS, Boulos M, Sinto SI, Caiaffa HH, Mendes CM, Oplustil CP, Sader H, Levy CE, Levin AS (1999). Nosocomial infections caused by multiresistant *Pseudomonas aeruginosa*. Infect Control Hosp Epidemiol.

[B5] Levin AS, Mendes CM, Sinto SI, Sader HS, Scarpitta CR, Rodrigues E, Sauaia N, Boulos M (1996). An outbreak of multiresistant *Acinetobacter baumanii *in a university hospital in Sao Paulo, Brazil. Infect Control Hosp Epidemiol.

[B6] Appleman MD, Belzberg H, Citron DM, Heseltine PN, Yellin AE, Murray J, Berne TV (2000). In vitro activities of nontraditional antimicrobials against multiresistant *Acinetobacter baumannii *strains isolated in an intensive care unit outbreak. Antimicrob Agents Chemother.

[B7] Hancock RE, Bell A (1988). Antibiotic uptake into Gram-negative bacteria. Eur J Clin Microbiol Infect Dis.

[B8] Clinical Laboratory Standards Institute (2005). Performance Standards for Antimicrobial Susceptibility Testing: Seventh Informational Supplement M100-S15.

[B9] Gales AC, Reis AO, Jones RN (2001). Contemporary Assessment of Antimicrobial Susceptibility testing Methods for Polymyxin B and Colistin: Review of Available Interpretative Criteria and Quality Control Guidelines. J Clin Microbiol.

[B10] Clinical Laboratory Standards Institute (2007). Performance Standards for Antimicrobial Susceptibility Testing: Seventh Informational Supplement M100-S17.

[B11] British Society for Antimicrobial Chemotherapy BSAC Disc Diffusion Method for Antimicrobial Susceptibility Testing, Version 4. http://www.bsac.org.uk/_db/_documents/version_4_january_2005_final_NH_april_2.pdf.

[B12] Comité de l'Antibiogramme de la Société Française de Microbiologie Recommandations du CASFM, Communiqué 2005 (Edition de Janvier 2005). http://www.sfm.asso.fr/doc.

[B13] National Committee for Clinical Laboratory Standards (2002). Methods for dilution antimicrobial susceptibility test for bacteria that grow aerobically. Approved standard M7-A5.

[B14] Package Insert (2003). BD BBL sensi-Disc Antimicrobial SusceptibilityTest Discs.

[B15] National Committee for Clinical Laboratory Standards (1981). Development of In Vitro Susceptibility Testing Criteria and Quality Control Parameters: Approved Standard. NCCLS M23-A2.

[B16] Jones RN, Anderegg TR, Swenson JM (2005). Quality Control Working Group. Quality control guidelines for testing gram-negative control strains with polymyxin B and colistin (polymyxin E) by standardized methods. J Clin Microbiol.

[B17] Tenover FC, Arbeit RD, Goering RV (1997). How to select and interpret molecular strain typing methods for epidemiological studies of bacterial infections: a review for healthcare epidemiologists. Molecular Typing Working Group of the Society for Healthcare Epidemiology of America. Infect Control Hosp Epidemiol.

[B18] Pitt TL, Sparrow M, Warner M, Stefanidou M (2003). Survey of resistance of *Pseudomonas aeruginosa *from UK patient with cystic fibrosis to six commonly prescribed antimicrobial agents. Thorax.

[B19] Schulin T (2002). *In vitro *activity of the aerolized agents colistin and tobramycin and five intravenous agents againts *Pseudomonas aeruginosa *isolated from cystic fibrosis patients in southwestern Germany. J Antimicrob Chemotherapy.

[B20] Bratu S, Quale J, Cebular S, Heddurshetti R, Landman D (2005). Multidrug-resistant *Pseudomonas aeruginosa *in Brooklyn, New York: molecular epidemiology and in vitro activity of polymyxin B. Eur J Clin Microbiol Infect Dis.

[B21] Landman D, Bratu S, Alam M, Quale J (2005). Citywide emergence f Pseudomonas aeruginosa strains with reduced susceptibility to polymyxin B. J Antimicrob Chemother.

[B22] Tan TY, Ng LS (2006). Comparison of three standardized disc susceptibility testing methods for colistin. J Antimicrob Chemother.

[B23] Hogardt M, Schmoldt S, Gotzfried M, Adler K, Heesemann J (2004). Pitfalls of polymyxin antimicrobial susceptibility testing of Pseudomonas aeruginosa isolated from cystic fibrosis patients. J Antimicrob Chemother.

[B24] Nicodemo AC, Araujo MR, Ruiz AS, Gales AC (2004). *In vitro *susceptibility of *Stenotrophomonas maltophilia *isolates: comparison of disc diffusion, Etest and agar dilution methods. J Antimicrob Chemother.

[B25] Tan TY, Ng SY (2007). Comparison of Etest, Vitek and agar dilution for susceptibility testing of colistin. Clin Microbiol Infect.

[B26] Goldstein FW, Ly A, Kitzis MD (2007). Comparison of Etest with agar dilution for testing the susceptibility of Pseudomonas aeruginosa and other multidrug-resistant bacteria to colistin. J Antimicrob Chemother.

[B27] Arroyo LA, Garcia-Curiel A, Pachon-Ibanez ME, Llanos AC, Ruiz M, Pachon J, Aznar J (2005). Realiability of the Etest-method for detection of colistin resistance in clinical isolates of *Acinetobacter baumannii*. J Clin Microb.

